# Effects of Concentrate Feed Starch Source Offered Twice a Day on Feed Intake and Milk Production of Cows During the Early Postpartum Period

**DOI:** 10.3390/ani14243622

**Published:** 2024-12-16

**Authors:** Rodrigo I. Albornoz, Victoria M. Russo, Christie K. M. Ho, Khageswor Giri, Michael S. Allen, Adam L. Lock, William J. Wales, Matthew I. Knight

**Affiliations:** 1 Agriculture Victoria Research, Department of Energy, Environment and Climate Action, Ellinbank, Victoria 3821, Australia; rodrigo.albornoz@dairyaustralia.com.au (R.I.A.); vicmrusso@gmail.com (V.M.R.); , matthew.knight72@gmail.com (M.I.K.); 2 Agriculture Victoria Research, Department of Energy, Environment and Climate Action, Bundoora, Victoria 3083, Australia; khageswor.giri@agriculture.vic.gov.au; 3 Department of Animal Science, Michigan State University, East Lansing, MI 48824, USA; allenm@msu.edu (M.S.A.); allock@msu.edu (A.L.L.)

**Keywords:** starch fermentability, early postpartum, milk production, feed intake

## Abstract

This experiment determined the effects of two different starch sources when offered twice a day to cows during the early postpartum period (1 to 23 d postpartum, treatment period) on dry matter intake (DMI), feeding behavior, and milk production. The subsequent effects on milk production in the carryover period (24 to 72 d) where cows received a common diet (grazed perennial ryegrass pasture plus concentrate supplements) were also measured. Thirty-two multiparous dairy cows were offered concentrate feed (8 kg DM/d) containing 5 kg DM of crushed wheat grain or ground corn grain (7 h in vitro starch digestibility of 65.8% and 58.8%, respectively). At each milking (morning and afternoon), cows were offered half of the concentrate feed, and upon return to their individual stalls, they were offered perennial ryegrass pasture silage (56.2% neutral detergent fiber (NDF), 39.8% acid detergent fiber (ADF), 16.6% crude protein (CP)) at 130% of their expected daily intake. Dry matter intake, milk yield, and body weight were recorded daily. Blood and milk sampling, body condition score, and feeding behavior measurements were performed weekly during the treatment period. During the carryover period, milk sampling and body condition score measurements were conducted on a weekly and fortnightly basis, respectively. In the treatment and carryover periods, there was no significant effect of starch source treatment on DMI, milk yield, milk composition, change in body weight, or body condition. Similarly, the starch source did not affect the concentrations of blood markers of energy status or inflammatory response. Despite differences in the in vitro starch fermentability between treatments, the starch source did not significantly affect production responses. It is possible that the elevated NDF and ADF concentrations of the forage offered during the treatment period, the limited difference in starch fermentability between treatments, and the temporal supply of fuels to the liver when starch sources were offered twice a day may have offset the effects of the type of starch source on DMI and production via physical signals associated with rumen fill and distention.

## Simple Summary

The postpartum period is a metabolically stressful time for dairy cows. During this period, cows face suppressed feed intake and a series of stressors that challenge the cow’s ability to adapt to lactation. Previous studies have shown that offering cows a total mixed ration diet containing a slowly fermentable starch source during the early postpartum period increases their feed intake when compared with that for a faster fermentable starch source. This leads to an immediate increase in milk production that carries over to later in lactation. These cows also exhibit an improved energy balance and decreased metabolic stress. However, the effects of feeding starch sources with differing fermentability during the early postpartum period in pasture-fed dairy systems are unknown. This experiment determined the effects of feeding starch sources with contrasting in vitro fermentability twice a day with perennial ryegrass silage as the forage source on feed intake, milk production, body reserves, feeding behavior, and markers of metabolic stress.

## 1. Introduction

The early postpartum period (~first 3 weeks postpartum) is a metabolically stressful time for dairy cows. During this period, cows face a series of management and environmental stressors that can challenge the cow’s ability to adapt to their new production environment. Some of these metabolic changes are characterized by a decrease in blood insulin secretion, an increase in insulin resistance by tissues, increased lipolysis, and increased inflammatory response [1]. In addition, suppressed dry matter intake (DMI) and increased milk energy output by cows during the early postpartum period predisposes these animals to a negative energy and nutrient balance and is associated with decreased productivity, health, and reproductive performance subsequent to the postpartum period [2,3,4]. This energy and nutrient deficit further exacerbates the mobilization of body reserves and increases the supply of fuels (e.g., free fatty acids) available for oxidation in the liver that can promote satiety [5].

During the early postpartum period, the cow’s requirements for glucose and glucose precursors for milk production and other bodily functions are increased. One strategy that may be used to increase the energy density and supply of glucose and glucose precursors for the cow is through the provision of dietary starch. However, the source of starch can affect DM and energy intake, as well as milk production [6]. The ruminal starch fermentability of grains varies with grain type and variety, processing and conservation methods, diet characteristics, and likely, temporal grain intake rate and digesta passage rate. Increasing the fermentability of starch increases the production and supply of propionate to the liver, promoting the oxidation of fuels and an increase in energy to the liver that triggers a satiety signal suppressing DMI in cows in early postpartum [5]. Hypophagic effects of propionate are more important during the early postpartum period when the mobilization of body reserves is increased and the pool of fuels available for oxidation in the liver is increased compared to that in later stages of lactation [1]. This mechanism of control of feed intake is known as the Hepatic Oxidation Theory [7].

Increasing DMI in the early postpartum period has been regarded as one of the most important factors to improve the energy balance in dairy cows [8]. However, diet characteristics and the type of feed determine the type, amount, and temporal absorption of fuels supplied to the cow and the effects on DM and energy intake. Furthermore, the type and amount of energy sources fed to cows during the early postpartum period can elicit carryover effects on metabolism and milk production [6,9]. Evidence from total mixed ration (TMR) production systems suggests that feeding cows a starch source with moderate ruminal fermentability (e.g., dry ground corn) as opposed to a highly fermentable starch source (e.g., high-moisture corn) during the early postpartum period can increase DM and energy intake and increase milk production during the early postpartum period [6]. Positive carryover effects on milk production in the subsequent phase of lactation when all cows received a common diet were also observed [6]. While the mechanisms involved in driving the carryover effects in that study are not fully understood, the authors hypothesized that the inflammatory response elicited by treatments during the early postpartum period may have played a role [10].

The effects of diet starch fermentability on early-postpartum cows in pasture-based grazing systems are unknown, along with whether there are any benefits on milk production that extend into the subsequent phase of lactation. In pasture-based grazing systems, cows normally consume concentrate feeds within minutes at milking (i.e., twice per day: morning and afternoon) and have access to the pasture upon returning to their paddock. This production system differs from a TMR system where cows have access to a mixed ration throughout the day, leading to a more staggered pattern of DM and nutrient intake. It would be expected that the timing of concentrate intake and, therefore, nutrient digestion and absorption rates and the effects on cow metabolism will differ in each system. Specifically, there would be more pronounced effects of treatments in cows offered concentrate feed at each milking (i.e., grazing cows) in the minutes and first hours post-concentrate intake. This contrasts with a less pronounced but more continuous effect throughout the day in a TMR system. Therefore, grazing cows during the early postpartum period may benefit from diets containing starch sources with moderate fermentability (e.g., dry ground corn grain) rather than highly fermentable ones (e.g., crushed wheat grain), promoting forage and energy intake and improving production during the early postpartum period and later in lactation.

The objective of this experiment was to determine the effects of the concentrate starch source offered twice a day to dairy cows during the early postpartum period on DMI, milk production, and markers of energy balance and metabolic stress as well as the carryover effects of treatments on production once cows received a common diet. The hypothesis was that the provision of a starch with moderate fermentability characteristics (ground corn) would lead to increased DMI and milk production, improved energy balance, and reduced metabolic stress compared with a more fermentable starch (crushed wheat) when offered twice daily to cows consuming a pasture silage of moderate nutrient characteristics.

## 2. Materials and Methods

### 2.1. Animal Care

This experiment was conducted at the Agriculture Victoria Research Centre, Ellinbank, Victoria, Australia (38°14′ S, 145°56′ E) in accordance with the Australian Code of Practice for the Care and Use of Animals for Scientific Purposes [11]. Animal use was approved by the Animal Research and Extension Animal Ethics Committee of the Department of Energy, Environment and Climate Action, Victoria (AEC 2019-09, approval date: 23 June 2019). All cows were in good health at the commencement of the experiment. Standard farm health and reproductive protocols were maintained throughout the experiment. Table 1 details the incidences of animal health and metabolic disorders during the treatment and carryover periods.

### 2.2. Experimental Design and Treatments

Thirty-two multiparous Holstein–Friesian cows were used in a completely randomized design to evaluate the effects of two dietary treatments with 16 cows per treatment. Treatments were allocated to cows at random subject to treatment group balanced for body condition score (BCS) observed within one week before expected calving date (using an 8-point scale, where 1 = thin and 8 = fat [12]), calving date (within 3 d), and parity. A common close-up diet was fed 21 days prior to the expected parturition date. During this period, cows were allowed to graze a perennial ryegrass pasture for most of the day and housed overnight in a calving facility with free access to water and barley hay (42.3% neutral detergent fiber (NDF), 15.6% starch, and 9.2% crude protein (CP)) and offered a mixed ration once a day containing corn silage, oaten grass hay, vetch hay, dry ground corn and wheat grain, canola meal, and a mineral and vitamin mix (34.8% NDF, 13.2% CP, and 11.3 MJ/kg DM ME). Soon after calving, cows received a commercial cow drench (0.7% calcium, 0.2% magnesium, and 0.25% potassium) and were housed in a maternity pen with free access to water and vetch hay until they recovered from the calving event and deemed fit (e.g., actively eating, no clinical signs of health disorders) to receive the allocated concentrate feed treatment at milking the following morning. Within 24 h after calving, cows were recruited into the experiment, except for one cow recruited at 36 h post-calving.

Once recruited into the experiment, cows were housed in a common loafing facility and offered half of their daily concentrate feed treatment allocation (4 kg DM) at each milking (morning: 6.15 h and afternoon: 15.15 h) and then offered perennial ryegrass pasture silage at 130% of expected intake (based on their previous day’s intake) in individual stalls for 3.5 h during each morning and afternoon feeding session. Perennial ryegrass silage was fed to cows in the early postpartum period in this experiment to try to replicate the pasture proportion of the cow’s diet in a pasture-fed grazing system and provide consistency in nutrient characteristics during the experiment, in contrast to cut pasture, which is rapidly changing at that time of the year. Concentrate feed treatments contained either dry ground corn (CRN) or crushed wheat (WHT) grains as the main starch source, with the treatment period lasting from d 1 to d 23 postpartum. Concentrate feed treatments were formulated to be iso-starch (47%) and isoproteic (16%) and contained solvent-extracted canola meal, finely ground almond hulls, rumen buffer, mycotoxin binder, minerals, and vitamins, with the main starch source being either dry ground corn grain or dry crushed wheat grain (Table 2).

In the subsequent carryover period, all cows received a common concentrate feed (7 kg DM/d) twice daily at each milking (7.15 h and 16.15 h) and grazed perennial ryegrass pasture at an allowance of ~20 kg DM/d from 24 to 72 d postpartum to investigate carryover effects of treatments.

### 2.3. Data and Sample Collection

During the treatment period, amounts of feed offered and refused were recorded for each morning and afternoon feeding session, with refusals recorded after 15 min for concentrate treatments and after 3.5 h for pasture silage relative to their offering. During the carryover period, the amount of concentrate offered was measured daily.

Representative samples of diet ingredients (0.5 kg) offered during the treatment and carryover periods were collected on the same day of the week ±3 d relative to the day of calving (5, 12, 19, 26, 33, 40, 47, 54, 61, and 68 d postpartum). Morning and afternoon concentrate treatments and pasture silage samples were collected each day during the treatment period and composited by day. All samples and composites were stored at −20 °C for later analysis of DM content and nutrient composition. Samples of concentrate treatments and pasture silage refusals (0.5 kg) were collected weekly (5, 12, and 19 d postpartum ±3 d) after the morning and afternoon feeding sessions and composited by day for each cow before being stored at −20 °C for later analysis of DM content.

Cow body weight (BW) was measured via the DeLaval automatic weigh system 100 (DeLaval AB, Tumba, Sweden) twice daily upon exiting the dairy. Body condition was scored weekly by three trained assessors on a 1–8 point scale [12]. Feeding behavior data were recorded using RumiWatch collars (RumiWatch, Itin + Hoch GmbH, Liestal, Switzerland) fitted over two consecutive days prior to data collection starting at 5, 12, and 19 d ± 3 d relative to the day of calving during the treatment period. Feeding behavior data were summarized at 5 min intervals as described by Norbu et al. [13] for each morning and afternoon feeding session, summed together between sessions and then averaged across consecutive days. Data were analyzed using the RumiWatch manager 2 version 2.2.0.0 and RumiWatch converter software version 0.7.4.13.

Milk yield data was recorded daily using inline milk meters (DeLaval ALPRO milk metering system, Tumba, Sweden). For milk composition and somatic cell count (SCC) analyses, milk samples were collected in containers with preservative (Bronopol, D&F Control Systems, San Ramos, CA, USA) on the afternoons of 5, 12, 19, 26, 33, 40, 47, 54, 61, and 68 d ±3 d postpartum and the following mornings. These samples were sent to Herd Improvement Co-operative Australia Ltd. (Korumburra, Victoria, Australia) for analysis of fat, protein, and lactose by means of a mid-infrared milk analyzer (Bentley FTS; Bentley Instruments, Chaska, MN, USA). Energy-corrected milk (ECM), standardized to 4.0% fat and 3.3% protein, was calculated using Equation (1) [6]:

ECM = (12.95 × fat yield) + (7.65 × true protein yield) + (0.327 × milk yield)(1)

Fat corrected milk (FCM) was estimated using quantity of milk calculated on a 3.5% butterfat energy basis [6]. The following formula was used:

3.5% FCM (kg/d) = 0.4324 × milk (kg/d) + 16.216 × fat (kg/d)(2)

Blood samples were collected via coccygeal venipuncture in the mornings before feeding at 6, 13, and 20 d ±3 d postpartum. Blood was collected in vacutainers containing K_2_-EDTA and immediately spun (3000× *g* for 15 min at 5 °C) to harvest plasma, which was stored at −20 °C. An additional measurement of BCS and collection of blood samples for analyte concentration analysis were performed within a week before parturition to be used as a covariate for statistical analysis. Blood plasma samples were analyzed for non-esterified fatty acids (NEFA; Randox Laboratories Pty. Ltd., Blackburn, UK), β-hydroxy butyrate (BHB) [14], glucose (Thermo Trace Pty. Ltd., Scoresby, Victoria, Australia), triglycerides (Thermo Trace Pty. Ltd., Scoresby, Victoria, Australia), haptoglobin [15], bilirubin [16], and albumin [17] concentrations using a variety of endpoint and kinetic (enzymatic activity) assays performed by Regional Laboratory Services, Benalla, Victoria, Australia.

### 2.4. Sample Analysis

Feed DM concentrations were determined by oven drying at 105 °C for 48 h twice per week for pasture silage during the treatment period and weekly for concentrate feeds throughout the experiment, with amounts offered adjusted accordingly. All rations were formulated to meet or exceed the cow’s predicted requirements for protein, minerals, and vitamins according to the National Research Council [18].

Samples of feed offered and refused were freeze-dried at −55 °C for at least 72 h and ground through a 1 mm screen. Feed samples and feed refusal composites samples were analyzed for DM content, ash, crude fat, ADF, NDF, CP, and starch concentration. During the treatment period, feed samples were also analyzed for metabolizable energy by Feed Central Pty. Ltd. (Charlton, Queensland, Australia). Differences in starch fermentability between CRN and WHT concentrate feed treatments were confirmed by 7 h in vitro starch digestibility analysis according to Goering and Van Soest [19] by Cumberland Valley Analytical Services, Waynesboro, PA, USA.

### 2.5. Statistical Analysis

Daily feed DMI during the treatment period and milk yield data during the treatment and carryover periods were averaged over the respective periods to achieve a mean value for each cow. Weekly milk composition, blood markers, BW, and BCS data were also averaged over the treatment period or carryover period, yielding a mean value for each cow for each period. Changes in BW and BCS during each period were calculated by final measurement (week 3 for treatment period and week 10 for carryover period) minus the initial measurement (week 1 for treatment period and week 3 for carryover period) for each period. These mean values for each cow were analyzed using analysis of covariance (ANCOVA) for the treatment period (from 1 to 23 d postpartum) and for the carryover period (from 24 to 72 d postpartum) separately. The ANCOVA model had starch source as treatment structure and cow as blocking structure. The pre-calving parity, BCS, and calving date data for each cow which had been used to balance treatment groups were also used as covariates in the ANCOVA analyses. During the carryover period, data from a cow fed the CRN concentrate were abnormal as she was recovering from an injury unrelated to the experiment. These data were excluded from the analyses. Histograms of residuals and plots of residuals versus fitted values were examined for normality of distribution with constant variance. Where necessary, response variables were logarithmically transformed prior to the final analyses. The treatment effect was considered statistically significant for *p*-values less than 0.05. All statistical analyses were undertaken using GenStat [20].

## 3. Results

Table 3 details the nutrient composition of the concentrate diet treatments, carryover concentrate, and perennial ryegrass pasture silage feed to cows. The ADF-to-NDF ratio for the perennial ryegrass silage was 0.70.

**Figure 1:**
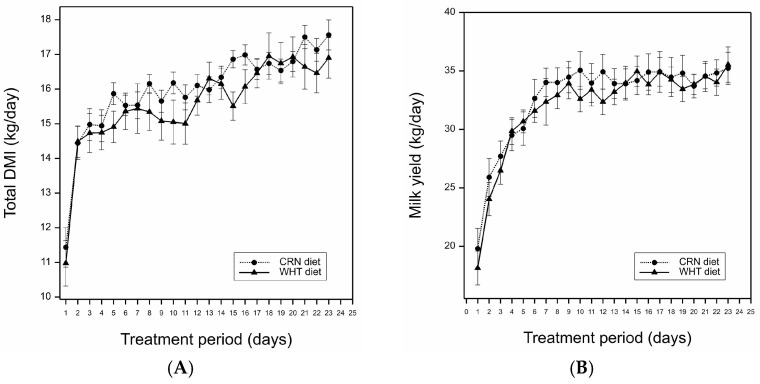
Average daily total dry matter intake (DMI; kg/day) (**A**) and milk yield (kg/day) (**B**) of cows fed a corn (CRN)- or wheat (WHT)-based concentrate in the treatment period (from 1 to 23 d postpartum). Error bars represent the mean standard error of the average daily total DMI and milk yield observed between cows for each treatment.

During the treatment period, the total (*p* = 0.28), concentrate (*p* = 0.21), and silage (*p* = 0.31) DMIs were not affected by the concentrate feed starch source (Table 4). Intake of NDF (*p =* 0.40), ADF (*p =* 0.32), and starch (*p =* 0.38) also did not differ between the treatments. Average daily total DMIs for cows receiving the CRN or WHT treatments during the treatment period are shown in Figure 1. Average BWs of cows during the treatment period differed between treatments (666 kg vs. 693 kg for CRN and WHT, respectively; *p* = 0.03), but the treatments did not affect BW changes during the treatment period (*p* = 0.13). Over the treatment period, the treatments did not affect the average BCS (*p* = 0.62) or BCS change (*p* = 0.42).

Cows fed the CRN treatment had greater rumination time when compared with that of cows fed the WHT treatment (81 min/d vs. 67 min/d; *p* = 0.03). Meanwhile, cows fed the WHT treatment spent more time idling compared with cows fed the CRN diet (94 min/d vs. 66 min/d; *p* = 0.01). The milk yield of cows on the CRN diet was 0.8 kg/cow higher than that of cows on WHT during the treatment period, but the difference was not statistically significant (*p* = 0.60). In addition, there were no significant effects of the starch source in the concentrate feeds on yields of ECM (*p* = 0.79), FCM (*p* = 0.80), fat (*p* = 0.57), protein (*p* = 0.83), or lactose (*p* = 0.65) during the treatment period (Table 4). Concentrations of blood plasma markers of energy status (BHB, NEFA, glucose, and triglycerides) and metabolic stress (haptoglobin, bilirubin, and albumin) were not affected by treatment (*p* > 0.05; Table 4).

During the carryover period, the starch sources offered during the treatment period did not affect body reserves, milk yield, or composition (Table 5). Specifically, the treatments did not affect cow BW (*p* = 0.62), BW change (*p* = 0.20), BCS (*p* = 0.31), or BCS change (*p* = 0.27) during the carryover period. Similarly, yields of milk (*p* = 0.87), ECM (*p* = 0.91), FCM (*p* = 0.89), fat (*p* = 0.92), protein (*p* = 0.98), and lactose (*p* = 0.96) during the carryover period were not affected by the starch source offered during the treatment period.

## 4. Discussion

Previous research conducted in TMR systems suggests that feeding starch sources of moderate fermentability (i.e., dry ground corn) to cows during the early postpartum period can increase DMI and decrease negative energy balance compared with those of cows that receive a highly fermentable starch source (i.e., high-moisture corn) [6,21]. Cows offered the less-fermentable starch source also had higher milk production in the early postpartum period and after this period when all cows received a common diet [6]. Corn grain contains starch with moderate ruminal fermentability compared with the more rumen-fermentable starch from wheat grain [22] and should allow for increased post-ruminal digestion of starch and differential supply of glucose and glucose precursors. The rate of starch fermentation in the rumen can modulate the production of volatile fatty acids, such as propionate, and the supply of these fuels to the cow. The literature suggests that increased ruminal production of propionate and its uptake by the liver can increase the hepatic oxidation of fuels and trigger a satiety signal that suppresses feed intake [1,23]. Therefore, in this study we hypothesized that early-postpartum cows that consumed the CRN treatment would have greater DM and energy intake, increasing the supply of energy to the cow and minimizing the negative energy balance and metabolic stress, compared with those of cows offered the WHT treatment.

There were no main effects of the starch source on the concentrate, forage, or total DMIs during the treatment period, nor milk yield in the treatment and carryover periods (Table 4 and Table 5), and thus, the hypothesis is not supported. Although the 0.8 kg/d increase in milk yield for cows fed the CRN diet was a numerical rather than statistically significant increase, it may still be biologically meaningful and valuable for farmers in their decision-making [24].

The lack of treatment effects on DMI and milk production compared with previous results [6] could relate to (1) the fill effects of the forage used in this study, (2) the limited difference in 7 h in vitro starch fermentability and interactions between rumen starch fermentability and forage quality, (3) the temporal supply of fuels derived from ruminal starch fermentability, and (4) the limited access to the forage source in the current study due to the experimental design. A difference of 2.2% in CP concentration between the CRN and WHT concentrates was present, not by design, resulting in an average difference in CP DM intake of 0.16 kg DM/cow/d. However, our findings do not suggest that this difference in CP concentration confounded the expected effects of starch source treatments, and therefore, it is not considered a primary factor influencing treatment outcomes.

Previous studies have reported that the filling effects of forages with high NDF concentrations limit DMI and decrease animal performance [25,26]. For example, Rockwell and Allen [27] reported that TMR diets containing starch sources with different fermentability (dry ground corn vs. high-moisture corn) did not affect DMI. The authors suggested that the physical effects from ruminal distention associated with the high forage NDF concentration in the TMR diet were the dominating signals controlling the cow’s DMI rather than metabolic signals associated with starch fermentability. These results suggest that forage quality, and particularly NDF concentration, play an important role in determining the extent to which metabolic signals associated with starch fermentability control feed intake and production in cows during the early postpartum period in both TMR and pasture-based systems.

The difference in in vitro starch fermentability between treatments fed during the treatment period was lower than that reported by Albornoz and Allen [6]; 7% vs. 17.8%, respectively. It is possible that in our study, the starch fermentability of the CRN and WHT were similar enough to mitigate any treatment effects on DMI. In addition, there was only a ~2 kg DM increase in daily DMI between the first and third weeks postpartum, independent of treatment (Figure 1). This limited increase in DMI was likely associated with the elevated concentrations of NDF and ADF (56.2% and 39.8%, respectively) of the perennial ryegrass silage offered to cows during the treatment period, which may have offset any potential metabolic effects of starch source on DMI.

Another reason contributing to the lack of significant treatment effects in our study may relate to the temporal production and supply of fuels to the liver when starch sources are offered twice a day as was the case in our study, in comparison to cows offered starch sources in a TMR throughout the day. In the current study, cows consumed their concentrate feed treatments within 15 min, likely promoting a rapid production and uptake of fuels that can decrease the mobilization of body reserves and circulating NEFA. A rapid reduction in circulating NEFA and the supply of acetyl CoA for oxidation in the liver can reduce hypophagic effects associated with hepatic oxidation [1,28].

In our study, the cows also had limited access to pasture silage during the treatment period (3.5 h during each morning and afternoon feeding session) compared with cows on TMR systems that have access to feed for most of the day. However, the feeding behavior data confirmed that the cows only spent ~4.3 h/d consuming pasture silage (269 min/d vs. 250 min/d for CRN and WHT, respectively), suggesting that cows were given sufficient time to consume the forage, as eating time did not approach the 7 h in which the feed was offered to the cows. Moreover, cows in both treatment groups had more than 1 h idling time during the 7 h period when the pasture silage was offered, indicating that cows had sufficient time to continue eating if hungry. These results are consistent with a previous study that showed that when offering feed for ab libitum consumption to cows in mid-lactation with a consistent feeding time, access to feed can be limited to 8 h daily with no adverse effects on performance [29].

Despite the absence of differences in forage DMI, cows offered WHT treatment ruminated on average 14 min/d less than cows fed the CRN treatment (*p* = 0.03). It is possible that the increased starch fermentability in WHT may have led to a differential ruminal environment and rumen function compared with cows that for receiving the CRN treatment. Souza et al. [30] conducted a review of the literature on cows in TMR systems and reported a positive association between rumen pH and rumination time. This suggests that a possible decrease in rumen pH by cows receiving the more fermentable WHT treatment led to a decrease in rumination compared with that of cows receiving the CRN treatment during the early postpartum period. However, rumination time outside of the forage feeding period (7 h/d) was not recorded in our study, and those initial differences may have diminished over time. Further supporting this notion, we did not observe differences in production between treatments, nor more importantly, in milk fat yield or fat concentrations, which are related to rumination behavior [30].

Blood markers of energy (NEFA, BHB, glucose, and triglycerides) and metabolic stress (haptoglobin, albumin, and bilirubin) were measured to determine whether different starch sources modulated the cows’ ability to metabolically adapt to the postpartum condition. Treatments did not affect NEFA, BHB, glucose, and triglyceride concentrations. Previous studies have reported that elevated levels of NEFA and BHB concentrations are prevalent with increased risk of metabolic disease and subclinical ketosis [31]. Some cows were diagnosed with hyperketonemia and metritis during the treatment period (Table 1). However, incidences of hyperketonemia and metritis recorded in this experiment were within the reported prevalence rates for dairy cows in the first two months of lactation [32]. In addition, the treatments did not affect the plasma concentrations of haptoglobin, albumin, and bilirubin, suggesting that the metabolic adaptation of cows to the early postpartum conditions was irrespective of the starch source offered in the concentrate feed.

Overall, including a starch source of moderate fermentability, such as corn grain, in concentrate feed offered to cows twice a day in the early postpartum period did not significantly affect DMI or milk production in the early postpartum period or in the subsequent stage of lactation, compared with cows offered a more fermentable starch source such as wheat grain when offered a forage source with moderate nutrient characteristics. Furthermore, cows metabolically adapted to the postpartum conditions independently of the main starch source in the concentrate feed. The dry matter intake of dairy cows is controlled by a continuum of physical (e.g., gut fill/rumen distention) and metabolic signals (e.g., hepatic oxidation), and one signal can dominate over the other at different times of the day depending on diet, feed quality, and the cow’s stage of lactation. The results from this experiment and the literature suggest that the elevated NDF concentration of the forage fed in this experiment likely exerted superior control over the forage and total DMI of cows compared with the metabolic signals associated with the starch source offered in the diet. In addition, the limited difference in starch fermentability between the starch sources used in this study and the effect of the temporal supply of fuels to the cow when concentrate feed is offered twice a day may have contributed to the decreased treatment effects. Future experiments investigating the impact of slow and fast fermenting starch sources on feed intake and milk production in early postpartum in pasture-based dairy systems should consider the use of forage sources of higher nutrient characteristics, such as a legume hay, that may decrease the physical signals from rumen distention and allow for increased DMI.

## 5. Conclusions

In this experiment, feeding cows in early postpartum twice a day a concentrate that contained dry ground corn or dry crushed wheat, where there was little difference in in vitro starch fermentability, resulted in no significant differences in feed intake, milk production, or changes to body reserves in this period or later in lactation. In addition, the treatments did not affect blood markers of energy status and metabolic stress, suggesting that the cows adapted to the postpartum conditions independent of the starch source offered. Our results and previous evidence suggest that the combination of high-NDF perennial ryegrass silage in the early postpartum period, the limited difference in the starch fermentability of the treatments, and the temporal supply of fuels when cows were offered the starch sources may have offset the anticipated differences in feed intake associated with the starch fermentability of concentrate treatments. Therefore, further research is required to fully elucidate the impact of feeding starch sources with contrasting ruminal fermentability to cows in the early postpartum period when offered a forage source more representative of the quality of a temperate pasture during vegetative stages.

## Figures and Tables

**Table 1:** Number of animal health incidences during the treatment and carryover periods.

Health Event	Treatment Period (1–23 d Postpartum)		Carryover Period (24–72 d Postpartum)	
	CRN ^1^	WHT ^1^	CRN ^1^	WHT ^1^
Mastitis	4	1	1	2
Metritis	3	1	1	0
Ketosis	0	5	0	0
Lameness	0	1	0	1
Udder edema	0	0	1	0

^1^ CRN = corn grain-based concentrate, WHT = wheat grain-based concentrate.

**Table 2:** Composition of concentrate feed treatments.

Ingredients (% DM)	Concentrate Diets ^1^	
	CRN	WHT
Wheat grain	-	67.9
Corn grain	65.6	-
Canola meal (solvent extracted)	21.1	18.2
Milled almond hulls	8.6	9.3
Mineral-vitamin mix ^2^	3	3
Limestone	0.9	0.9
AcidBuf ^3^	0.5	0.5
Magnesium Oxide	0.2	0.2
Elitox ^4^	0.12	0.12

^1^ CRN = corn grain-based concentrate, WHT = wheat grain-based concentrate. ^2^ Mineral–vitamin mix contained on a DM basis: 25.6% NaCl, 10.0% Ca, 2.0% Mg, 2.0% P, 30 ppm of Co, 506 ppm of Cu, 20 ppm of I, 2220 ppm of Fe, 2080 ppm of Mn, 15 ppm of Se, 2030 ppm of Zn, 300 kIU/kg of vitamin A, 50 kIU/kg of vitamin D, and 1500 kIU/kg of vitamin E. ^3^ Calcareous marine algae (Acid Buf, Celtic Sea Minerals, Cork, Ireland). ^4^ Mycotoxin binder (Impextraco NV, Heist-op-den-Berg, Belgium).

**Table 3:** Nutrient composition of concentrate diet treatments, carryover concentrate, and perennial ryegrass silage.

Nutrient Parameter	Starch Source		Carryover Concentrate	Perennial Ryegrass Silage
	CRN ^1^	WHT ^1^		
% DM	92.6	93.4	90.5	88.5
Neutral detergent fiber (% DM)	14.4	14.4	11.7	56.2
Acid detergent fiber (% DM)	8.8	8.5	4.8	39.8
Crude protein (% DM)	16.3	18.5	14.2	16.6
Starch (% DM)	48	48.1	60.5	1.4
Ash (% DM)	7.3	8.7	9.3	10.7
In vitro starch digestibility, 7 h (% DM)	58.8	65.8	56.6	Not tested
Metabolizable energy (MJ/kg DM)	13.0	12.6	12.5	9.3

^1^ CRN = corn grain-based concentrate, WHT = wheat grain-based concentrate.

**Table 4:** Effects of concentrate feed starch source on feed intake, milk production, body reserves, feeding behavior, and blood metabolites during the treatment period (1 to 23 d postpartum).

	Starch Source		SED	*p*-Value
	CRN ^1^	WHT ^1^		
**Feed intake**				
Total DMI ^2^ (kg/d)	16.0	15.6	0.393	0.28
Concentrate DMI ^2^ (kg/d)	7.92	7.88	0.035	0.21
Silage DMI ^2^ (kg/d)	8.06	7.67	0.373	0.31
Neutral detergent fiber intake (kg/d)	5.63	5.45	0.208	0.40
Acid detergent fiber intake (kg/d)	3.88	3.73	0.147	0.32
Starch intake (kg/d)	3.92	3.90	0.020	0.38
**Consumed diet nutrient composition**				
Neutral detergent fiber (% DM)	35.2	34.5	0.005	0.16
Acid detergent fiber (% of DM)	24.3	23.6	0.004	0.08
Starch (% DM)	24.8	25.6	0.006	0.14
**Body energy reserves**				
Average post-calving BW ^3^ (kg)	666	693	11.3	0.03
Δ BW ^3^ at 3 weeks (Week 3–Week 1, kg)	−45.4	−55.7	6.6	0.13
Average post-calving BCS ^3^ (1–8 scale, units)	4.63	4.66	0.055	0.62
Δ BCS ^3^ (Week 3–Week 1, units)	−0.31	−0.35	0.051	0.42
**Feeding Behavior**				
Rumination time (min/d)	81.2	67.0	6.29	0.03
Eating time (min/d)	269	250	14.0	0.20
Idling time (min/d)	66	94	9.75	0.01
**Milk**				
Milk yield (kg/d)	32.7	31.9	1.56	0.60
ECM ^4^ (kg/d)	38.9	39.4	1.88	0.79
FCM ^4^ (kg/d)	39.6	40.1	2.03	0.80
Fat (kg/d)	1.57	1.62	0.091	0.57
Protein (kg/d)	1.03	1.04	0.045	0.83
Lactose (kg/d)	1.61	1.58	0.075	0.65
SCC ^4^ [1000/mL] (log_10_ SCC)	95.5 (4.98)	134.9 (5.13)	(0.461)	0.75
Fat (%)	4.86	5.13	0.188	0.17
Protein (%)	3.20	3.32	0.100	0.24
Lactose (%)	4.94	4.94	0.043	1.00
**Metabolic indicators**				
BHB ^5^ [mmol/l] (log_10_ BHB)	0.36 (−0.44)	0.40 (−0.39)	(0.142)	0.69
NEFA ^5^ (mmol/L)	1.05	1.22	0.137	0.23
Glucose (mmol/L)	3.39	3.54	0.090	0.10
Triglycerides (mmol/L)	0.12	0.13	0.007	0.58
**Stress indicators**				
Haptoglobin [g/L] (log_10_ haptoglobin)	0.022 (−1.64)	0.016 (−1.80)	(0.300)	0.58
Bilirubin (mmol/L)	6.37	5.67	0.421	0.11
Albumin (g/L)	34.5	35.0	0.731	0.47

^1^ CRN = corn grain-based concentrate, WHT = wheat grain-based concentrate. ^2^ DMI = dry matter intake. ^3^ BW = body weight and BCS = body condition score. ^4^ ECM = energy-corrected milk, FCM = fat-corrected milk, and SCC = somatic cell count. ^5^ BHB = β-hydroxy butyrate concentration and NEFA = non-esterified fatty acid concentration.

**Table 5:** Effects of concentrate feed starch source offered during the treatment period (1 to 23 d postpartum) on body reserves, milk production, and milk composition during the carryover period (24 to 72 d postpartum).

	Starch Source		SED	*p*-Value
	CRN ^1^	WHT ^1^		
**Body energy reserves**				
Average BW ^2^ (kg)	606	614	15.9	0.62
Average Δ BW ^2^ (Week 10–Week 3, kg)	−6.66	−19.66	9.79	0.20
Average BCS ^2^ (1–8 scale, units)	4.41	4.35	0.066	0.31
Average Δ BCS ^2^ (Week 10–Week 3, units)	−0.17	−0.23	0.056	0.27
**Milk**				
Milk yield (kg/d)	38.8	38.5	1.50	0.87
ECM ^3^ (kg/d)	40.8	40.7	1.55	0.91
FCM ^3^ (kg/d)	40.6	40.4	1.47	0.89
Fat (kg/d)	1.47	1.46	0.061	0.92
Protein (kg/d)	1.20	1.19	0.056	0.98
Lactose (kg/d)	1.96	1.96	0.076	0.96
SCC ^3^ [1000/mL] (log10 SCC)	39.8 (4.60)	15.5 (4.19)	(0.432)	0.36
Fat (%)	3.80	3.81	0.135	0.93
Protein (%)	3.09	3.09	0.066	0.96
Lactose (%)	5.05	5.08	0.046	0.51

^1^ CRN = corn grain-based concentrate, WHT = wheat grain-based concentrate. ^2^ BW = body weight and BCS = body condition score. ^3^ ECM = energy-corrected milk, FCM = fat-corrected milk, and SCC = somatic cell count.

## Data Availability

Data are available from the corresponding author upon reasonable request.
